# Quantifying post‐release behaviour of a critically endangered elasmobranch over two capture events using high‐resolution archival tag data

**DOI:** 10.1111/jfb.70542

**Published:** 2026-06-22

**Authors:** Danielle L. Orrell, Jamie Darby, Damien Haberlin, Alfonso Cohuo, Luke Harman, Thomas K. Doyle

**Affiliations:** ^1^ School of Biological, Earth and Environmental Sciences, University College Cork Cork Ireland; ^2^ MaREI Centre, Sustainability Institute University College Cork Cork Ireland; ^3^ School of Biological Sciences, University of Auckland Auckland New Zealand

**Keywords:** acoustic tracking, archival, batoid, biologging, elasmobranch conservation, electronic tracking, MiniPAT PSAT, satellite tagging

## Abstract

During catch‐and‐release angling, highly resident species are susceptible to multiple recapture events. Biologging offers the opportunity to study post‐release behaviour in detail; however, most studies have focused on a single capture‐release event. Using high‐resolution archival data and acoustic tracking, we present the post‐release behaviour of a single flapper skate (*Dipturus intermedius*) that was captured, tagged and recaptured 6 days later off the southwest coast of Ireland. As programmed, the archival satellite tag released 7 days after the initial capture event and 1 day after the second capture event, and the skate was monitored for an additional 212 days using an array of acoustic receivers. Analysis of the archival satellite tag dataset revealed atypical movements, characterised by increased mobility and vertical descents/ascents following release on both occasions. Generalised additive models corroborated that changes in depth and mobility were high after animal capture and release. Change point analysis identified that at 9 and 10 h after the first and second capture event, respectively, the skate's behaviour changed from increased mobility and rapid movements to more settled behaviour. The average integrated vertical movement (IVM) post‐capture, before the identified change points, was 441% higher than the mean hourly IVM outside this timeframe (5.15 versus 22.73 m/h). Acoustic tracking data corroborated that the skate remained near the capture area after both capture events. This study demonstrates the behavioural response of a flapper skate to two capture events and shows that, while capture led to 9–10 h of elevated activity, the skate remained in the capture area despite multiple capture events.

## 1 INTRODUCTION

Biologging has provided unparalleled insight into the behaviour of free‐living animals, their internal states and interactions within their surrounding environment (Watanabe & Papastamatiou, 2023). Biologging data have been used to explore topics such as animal energetics, foraging, migration and species interactions (Wilmers et al., 2015). And in turn, it has informed management, including stock assessments (Bacheler et al., 2009; Hightower & Pollock, 2013) and welfare assessments (Brijs et al., 2018), as well as spatial conservation measures (Hays et al., 2019). Biologging has also been used to study the post‐release behavioural responses of elasmobranchs across a range of contexts by relating swimming performance metrics to behavioural changes following release (Hoolihan et al., 2011; Mohan et al., 2020; Whitney et al., 2021). Whereby irregular behaviour can be identified based on changes in tailbeat frequency (Grainger et al., 2022), overall dynamic body acceleration (Binstock et al., 2023) or an increase or reduction in vertical depth use (Campana et al., 2009; Hoolihan et al. 2011), and subsequent recovery as a return to more ‘typical’ movement behaviours (Whitney et al., 2016). Opportunistic recapture of tagged animals can provide high‐resolution data to investigate catch‐and‐release (C&R) responses and, therefore, assess potential implications for species management.

Elasmobranchs are increasingly targeted for sport by recreational anglers, comprising approximately up to 6% of marine recreational angling catches (Freire et al., 2020). While the commercial retention and landing of several conservation‐listed elasmobranchs is prohibited under the common fisheries policy [Council Regulation (EC) 43/2009], C&R angling is permitted. Capture events are typically high‐stress and exhaustive and can lead to altered biochemical and behavioural responses (Danylchuk et al., 2014; French et al., 2015; Gallagher et al., 2017; Iosilevskii et al., 2022). Perceived sub‐lethal effects include reduced feeding ability and the energetic costs of injury, secondary infections and capture‐induced parturition (Adams et al., 2018; Borucinska et al., 2002), to lethal effects, such as delayed post‐release mortality (Mohan et al., 2020; Skomal, 2007). The severity of these sub‐lethal effects relates to factors including angling practices, time on deck, terminal gear considerations (rod‐and‐line strength, reel type, hook size and shape, etc.) and handling procedures (see review by McLoughlin & Eliason, 2008), as well as capture conditions (fished depth, water temperature, hooked location, fight times; Cooke & Suski, 2005; Danylchuk et al., 2014), and inherent species sensitivity (i.e., physiological scope, tolerance, reproductive condition; Mandelman & Skomal, 2009). Most C&R research has focused on teleosts and sharks, with few quantitative studies described for batoids (Batoidea) (Cameron et al., 2023; Skomal & Mandelman, 2012).

Flapper skates are an elasmobranch of global conservation concern (Loca et al. 2025) and are listed as critically endangered on the Irish Red List (Clarke et al., 2016) and the global IUCN Red List (Ellis et al., 2024). The flapper skate is a slow‐growing, long‐lived, large‐bodied benthic elasmobranch that is prone to taxonomic confusion; characteristics that make it vulnerable to extinction (Ellis et al., 2024). While prohibited from commercial harvest [Council Regulation (EC) 43/2009], recreational C&R angling across its remaining distribution is common and largely unregulated (Garbett et al., 2021). Mark–recapture datasets highlight the interannual site fidelity of flapper skate in both Scottish and Irish waters, as evidenced by repeated captures of the same individuals across multiple years (Little, 1995; Neat et al., 2015; Orrell et al., 2026). To date, one study has investigated the behavioural effects of C&R angling on flapper skate through recapture of skate equipped using archival tags (depth and temperature recordings logged every 2‐min) in the Loch Sunart to the Sound of Jura Marine Protected Area, in western Scotland (*n* tagged flapper skate = 21, *n* recaptures = 5, bathymetric depths = <290 m; Lavender et al., 2022). This study identified that immediately following release, tagged skate typically moved into deeper water and exhibited short periods (1–2 h) of low vertical activity. Additionally, overall vertical activity was ~38% higher in the 12 h following release compared to undisturbed activity. However, data were recorded every ~2‐min, and it was not possible to assess potential horizontal displacement as only archival depth data were available for movement analyses.

The study presented here examines the short‐term behavioural response of a flapper skate equipped with a high‐resolution (recor pop‐off satellite tag (recording data every second) and an acoustic tag to (i) investigate the vertical and horizontal movement response of a flapper skate to two capture events in a shallow water environment and (ii) quantify the time taken for the skate to return to its typical depth range and activity levels.

## 2 MATERIALS AND METHODS

### 2.1 Study site and the acoustic receiver array

**FIGURE 1:**
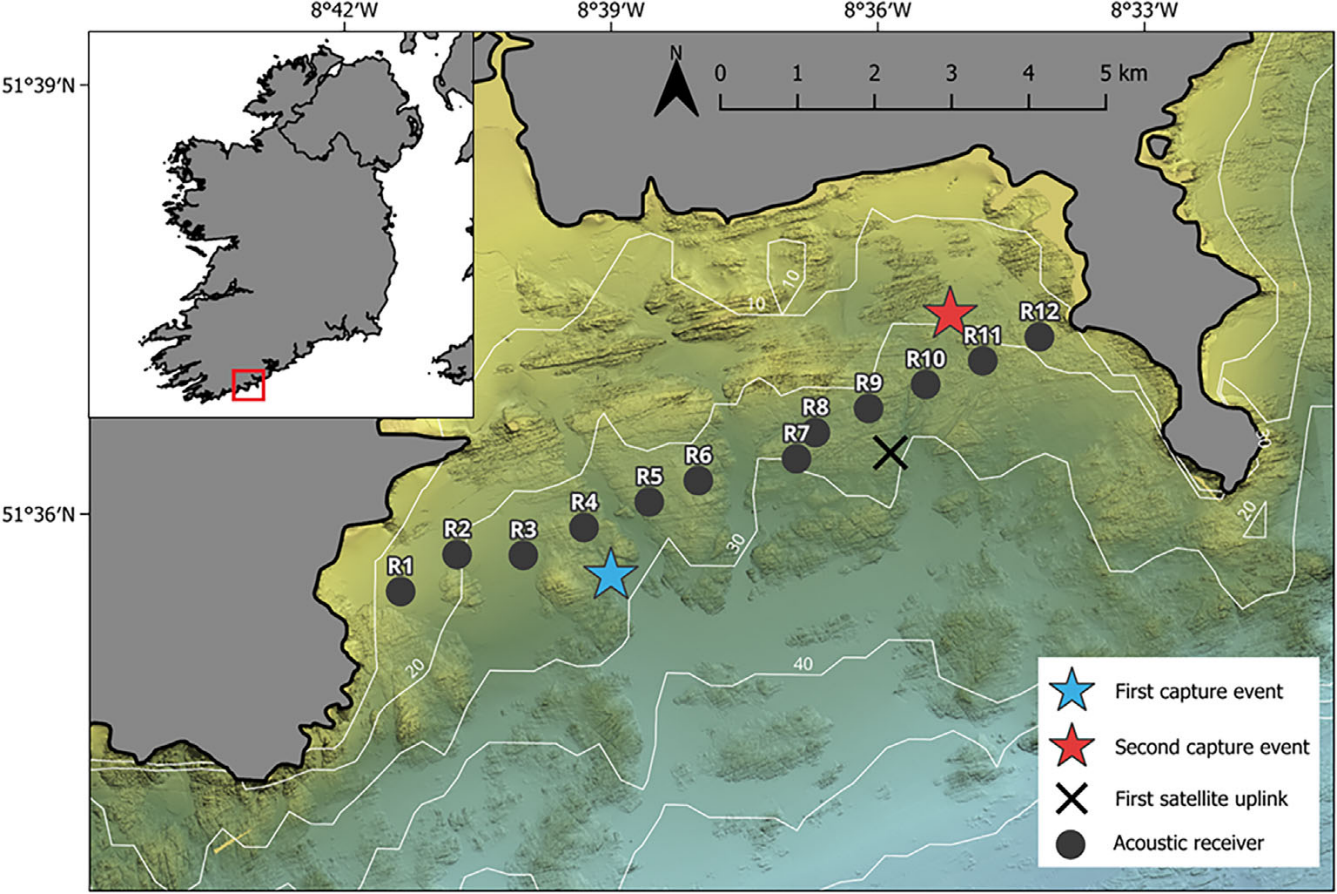
(Inset) Courtmacsherry Bay, Co. Cork (red box), situated on the southwest coast of the Republic of Ireland (51°36′ N, 8°37′ W). In the main figure, black circles denote the location of acoustic receiver stations (*n* = 12), with stars indicating the capture locations of the skate (first, red; second, blue) and the first satellite uplink from the MiniPAT tag (Argos class 3 position, location provided to an estimated error of <250 m) shown as a black cross. Topographic basemap downloaded from INFOMAR available from https://www.infomar.ie/data.

Courtmacsherry Bay, Co. Cork, in southwest Ireland (51°36′ N, 8°37′ W), is a shallow bay (depth = 10–40 m) predominantly composed of sand, mud and broken ground. Locally, there are four vessels engaged in recreational angling charters and marine tourism, and four inshore vessels involved in a variety of fishing activities, including trawling, netting and potting. In 2023, 12 acoustic receivers (VR2W = 10, VR2AR = 2, Innovasea Systems Inc., NS, Canada) were deployed between two adjacent headlands in Courtmacsherry Bay, Co. Cork, Ireland to form an acoustic gate (Figure 1). Receivers were spaced 800 m apart, as determined using a 24 h fixed range test (further details in Figure S1). Mooring lines comprised of an 11″ subsurface trawl float affixed to a 14 mm polypropylene rope, which terminated in an 80 kg chain anchor. Acoustic receivers were attached to the polypropylene rope 2–3 m below the trawl float to minimise acoustic shadowing. Receiver maintenance and download began on 8th February 2025. Therefore, the total acoustic tracking period in this study spans 219 days.

### 2.2 Ethical statement

The care and use of experimental animals complied with Irish animal welfare laws, guidelines and policies, as approved by the University College Cork Animal Experimentation Ethics Committee (AEEC) and the Irish Health Products Regulatory Authority (HPRA) under permit AE19130/P189. All procedures were carried out by licensed and trained personnel. Animal handling followed best‐practice guidelines for the focal species.

### 2.3 Capture, sampling and tag attachment

**FIGURE 2:**
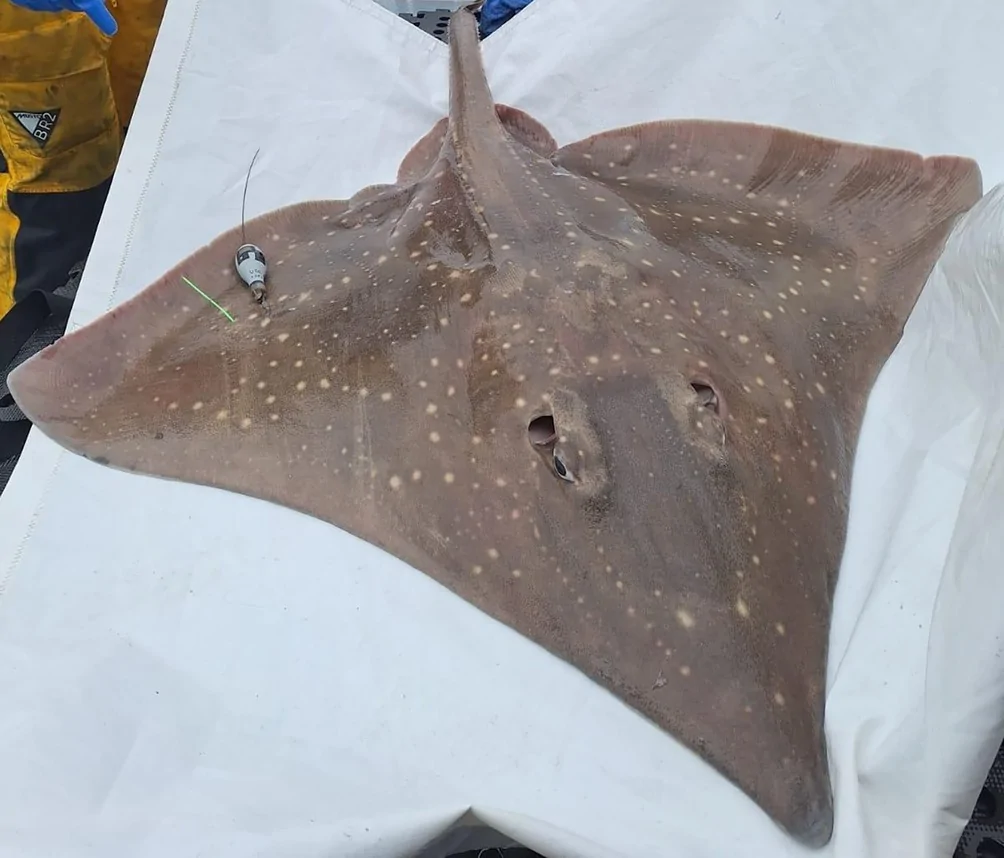
A female flapper skate (*Dipturus intermedius*) fitted with a pop‐off satellite transmitter (miniPAT; Wildlife Computers, Oregon, USA) prior to release.

A flapper skate was captured on the 4th of July 2024 at ~25 m depth in Courtmacsherry Bay using rod and line with a barbless 10/0 J‐hook after an 8‐min fight time. Once landed, the skate was inverted and placed on a 2 × 2 m lifting mat, and the spiracles were continuously irrigated with fresh seawater using a boat hose. Lidocaine (1 mL, concentration = 0.02 mg L^−1^) was injected at the acoustic tag implantation site. Sampling procedures also included taking a 3mL blood sample from the caudal vein using a 21G needle and collecting a small tissue sample from the first dorsal fin (posterior region, tip; 600–800 mg). After 3 min (the est. time taken for lidocaine to have a localised effect, James Thorburn *pers. comm*.), a V13‐1H acoustic transmitter (36 mm length, 11 g weight in air, 60–180‐s transmission delay, 152‐dB re 1μPa @1 m, 633‐d tag life; Innovasea Systems Inc., NS, Canada) was surgically implanted into the intraperitoneal cavity, and the incision was closed using two independent sterile sutures (3–0 monofilament absorbable, Ethicon Ltd., Livingston, UK). The skate was then inverted, and lidocaine (1 mL, concentration = 0.02 mg L^−1^) was injected into the satellite tag tether attachment site. To attach the satellite tag (118 mm length, 38 mm diameter, 61 g weight in air; MiniPAT, Wildlife Computers, Redmond, WA), a 1 cm incision was made in the wing to ease the application of a small titanium dart (45 mm length, 14 mm diameter, 3 g in air; Wildlife Computers, Redmond, WA) into the dorsal musculature. The dart was attached to the satellite tag using a 200‐lb. monofilament tether and stainless‐steel crimps (see Supplementary material S2 and Figure S2 for further information) (Figure 2). The incision was closed with a single independent sterile suture (3–0 monofilament absorbable, Ethicon Ltd., Livingston, UK). Sex was determined based on the absence of claspers. Any notable characteristics (e.g., scratches indicative of mating behaviour or scars, and pelvic swelling as described in Mawer et al. (2025)) were subsequently recorded, as well as total length (FL) and disc width (DW), which were measured over the curvature of the body (cm). An Inland Fisheries Ireland conventional tag was then implanted on the dorsal surface to aid in future identification. The animal was released after 18 min of processing at the capture location. Six days later, the project team incidentally recaptured the skate while targeting additional skates for satellite tagging. The recapture occurred on the 10th of July 2025, ~6 km from the initial capture site (as identified by the unique conventional tag ID and satellite tag), in ~18 m of water using a rod and line, following a 3‐min fight. On‐deck processing time was less than 5 min, after which the animal was released at the site of capture.

### 2.4 Satellite tag programming

The satellite tag (MiniPAT, Wildlife Computers Inc., WA, USA) was programmed to archive temperature (resolution = 0.05°C, accuracy = ± 0.1°C), depth (resolution = 0.5 m, accuracy = ± 1% of reading), light level (5 × 10–12 W cm^−2^ to 5 × 10–2 W cm^−2^), mobility and tag orientation (measured as acceleration on the z‐axis, Az) every second for 7 days. As described in Skubel et al. (2020), mobility is the row‐wise mean of the standard deviation (*σ*) of acceleration across three axes (*x*, *y* and *z*‐axes denoted as *A*_*x*_, *A*_*y*_, *A*_*z*_) sampled at 8 Hz. The standard deviation is calculated over a 3 s sliding window that advances by 1 s increments, and thereafter recorded every 1 s:

(1) $${\text{Mobility}}_{i}=\frac{\sum_{i=1}^{24}\sigma \left(A_{\mathrm{xi}}+A_{\mathrm{yi}}+A_{\mathrm{zi}}\right)}{24}$$

### 2.5 Movement analyses

Biologging data were analysed in R (v. 4.4.2; R Core Team, 2024). Satellite tag data were visualised and outliers or clearly erroneous readings were removed prior to further analyses. This constituted removing a section of consistently high mobility values (≥150) during the last 4 h of the deployment that may have occurred when the device released from the skate. Pressure‐derived tag depth was subtracted from publicly available local tidal gauge data (Union Hall; data hosted by the Irish National Tide Gauge Network/Marine Institute) to calculate animal depth relative to static sea level, described hereafter as absolute depth.

To investigate post‐release vertical movements, a rolling 1‐min mean of depth and mobility was calculated, and the data were subsampled to 1‐min intervals for further analyses. Integrated vertical movement (IVM) was calculated following the process described in Hays et al. (2012). IVM was calculated as the sum of the absolute differences in depth between data points (*n* and *n* + 1) per hour.

To further quantify changes in behaviour post‐release, two separate generalised additive models (GAMs) were fitted, with mobility and IVM as response variables. Mobility was modelled as a 10‐min rolling mean to account for issues of autocorrelation and overdispersion evident in the 1‐min equivalent. IVM was provided at 1‐h resolution. GAMs were fitted using the *mgcv* package (Wood et al., 2016); all models used the same set of descriptor variables. For further information on model fit, see Supplementary material S3 and Figures S3 and S4. A tensor product descriptor of time since capture (log transformed; “time_since_log” in the model formula below) and capture instance (“post_cap”) was constructed using a thin plate regression spline (basis = “tp”) and a random effect spline (basis = “re”), respectively. This approach enabled us to account for the potential confounding effects that the environment may have on these movement metrics. In this instance, tide height in metres (“tide”) and raw light levels recorded by the satellite tag (“LightLevel”) were included as thin plate regression splines with shrinkage (basis = “ts”) to account for the impact of tide phase and light availability on movements post‐release. However, while useful for looking at the impact of capture on movement independent of these environmental influences, it inversely allowed us to investigate the impact of these environmental covariates on movement metrics while accounting for the confounding effects of capture. To account for autocorrelation inherent in animal movement (i.e., serial) data, a corrective first‐order autoregressive error function was included in each model (e.g., Kane et al., 2020), following identification of autocorrelation between residuals. Both models were fitted with an exponential error structure with a log link, as variance in the response was greater than the square of the mean (following Salinas Ruíz et al., 2023). Model formulae were specified as follows:

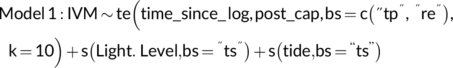




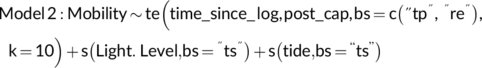

A change point analysis was then used to quantify the time taken for the skate to settle to lower mobility levels post‐capture using the *mcp* package (Lindeløv, 2020). Mobility was included as the response variable, time since capture (up to a limit of 32 h) as a descriptor and assuming disjoined independent slopes and a single changepoint in the data. The most recent capture instance (i.e., capture for tagging or secondary capture) was included as a random effect to account for any differences in response to either event.

Using the acoustic data, residency days were defined as the number of days the skate was detected at least twice within a 24‐h period during the 7‐day satellite and acoustic tracking period, and during the entire 219‐day acoustic tracking study period.

## 3 RESULTS

The flapper skate had no notable scratches (indicative of mating behaviour or interactions) or pelvic swelling (indicative of egg‐bearing). Given these external characteristics and the animal's size (TL = 169 cm, DW = 124 cm), it is likely that it was an immature female, in line with length‐maturity estimates by Régnier et al. (2021) and Thorburn et al. (2023).

### 3.1 Vertical and horizontal movements

**FIGURE 3:**
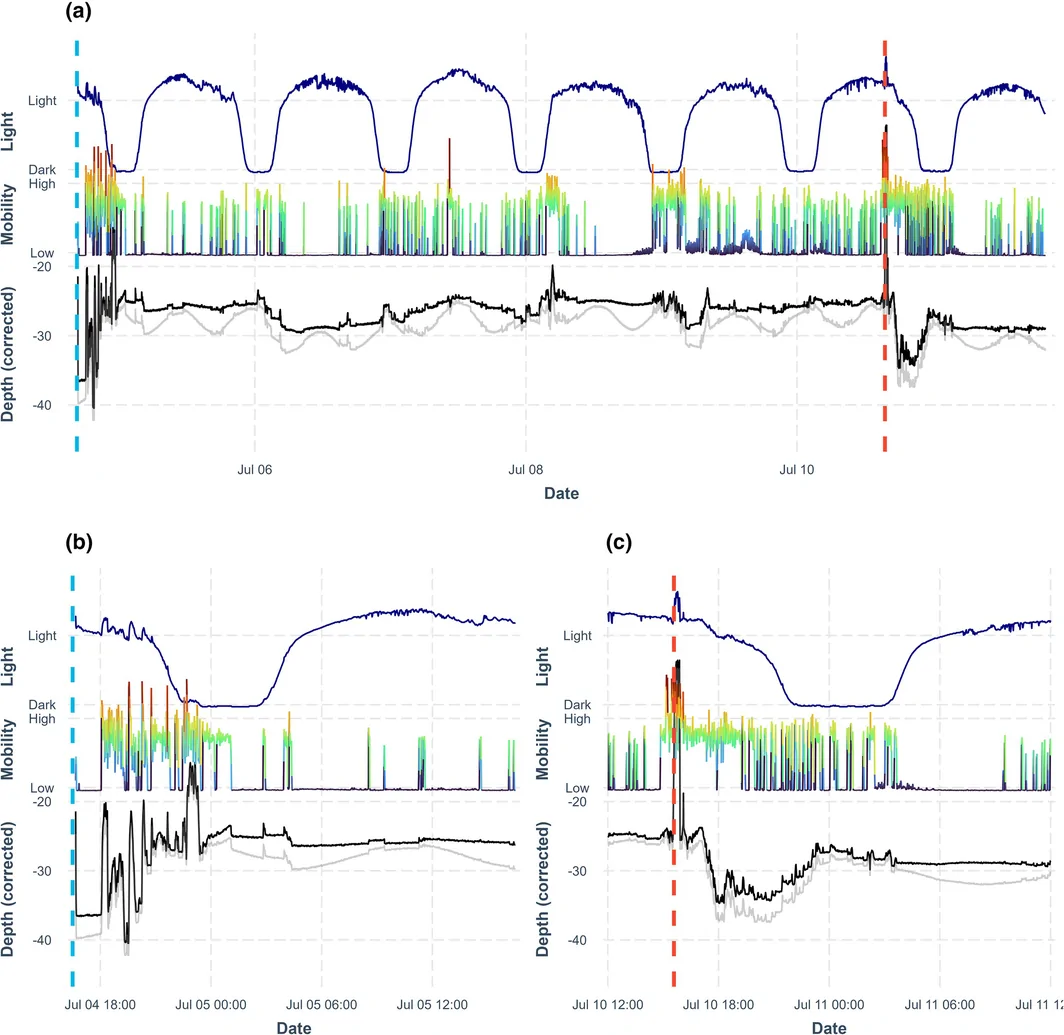
Time series of light, mobility and depth from the miniPAT for (A) the full tagged duration, (B) following the first capture/tagging event, (C) following secondary capture. Dashed vertical lines represent tagging (blue) and secondary capture (red). Light and mobility are presented on arbitrary scales of dark/light and low/high, respectively, to effectively visualise day and night times and periods of activity versus inactivity. Depth is presented in metres, the black line is corrected for tide and the grey line is uncorrected.

Over the seven‐day satellite tag attachment period, more than 600,000 depth data points were recorded, with tag depth logged every second. After the initial capture event, the animal descended to and remained at a depth of 36.5 m for a period of just over 1 h (IVM = 6.4 m/h) with consistently low mobility (median = 6, IQR = 5.9–6.1) (Figure 3a,b) and was not detected in the acoustic array area. After this, the skate engaged in high‐energy movement for 8 h between depths of 14.3 m and 40.5 m (median IVM = 35.2 m/h, IQR = 7.1–61.4 m/h; median mobility = 75.4, IQR = 7.2–88.1). The skate was detected by the array for the first time 6 h after the initial capture event and was detected sporadically on receivers R5 and then R6 6–9 h post‐capture, while in this high‐energy movement state. Nine hours post‐capture, there was a reduction in activity and occupation of depths between 19.8 m and 29.6 m until the next capture event ~6 days later (median IVM = 4.0 m/h, IQR = 3.4–5.6 m/h; median mobility = 6.8, IQR = 6.2–11.8) (Figure 3c) with intermittent detections on receivers R5 and R6 during this time. Immediately after the second capture event the skate did not exhibit a return to depth and subsequent static period as seen after the first capture event; instead, the skate began to engage in high‐energy movement (median IVM = 35.2 m/h, IQR = 7.1–61.4 m/h; median mobility = 69.6, IQR = 6.5–88.6), swimming between depths of 18.8 m and 34.7 m. The skate was detected on receiver R5 41 min after the second capture event and sporadically detected during the high‐energy movement phase. After 10.6 h, it again its reduced activity and vertical movements for the remainder of the deployment (1.4 days, median IVM = 3.9 m/h, IQR = 3.0–5.1 m/h, median mobility = 6.3, IQR = 6.1–7.5) and remained between 26.3 m and 29.6 m depth (at which point the tag released from animal at the predetermined 7‐days post deployment).

**FIGURE 4:**
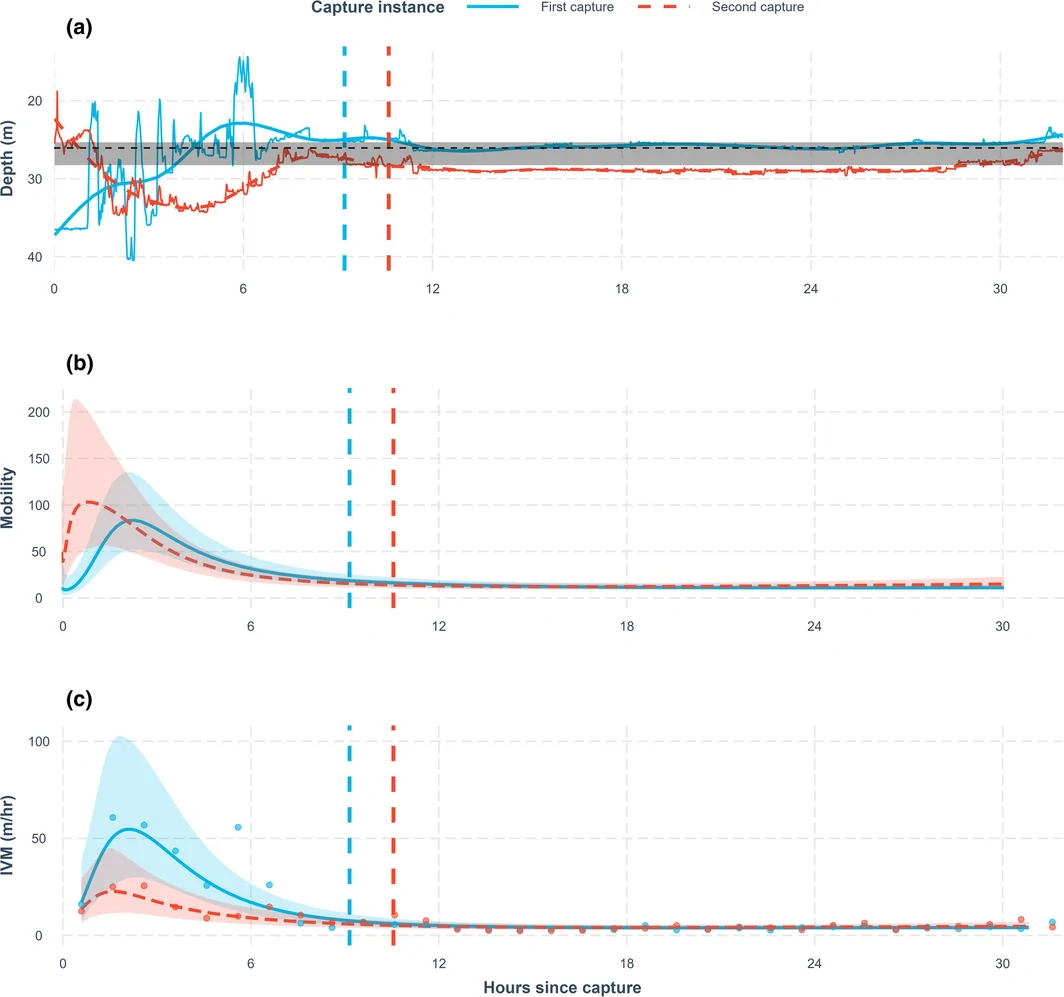
(A) Rolling 1‐min mean of depth (corrected for tide) for 32 h post‐tagging event (blue) and after the second capture event (red). The smooth lines for each trend are constructed using a basic thin plate regression spline with 20 knots. The horizontal dashed line is the median depth of the skate across the 7‐day period, and the shaded area is the inter‐quartile range. (B, C) Thin plate regression splines of mobility (B) and IVM (C) against hours since capture, each fit using a generalised additive model (GAM). Blue smooths correspond to the period post‐tagging, and the red smooths correspond to the period post‐secondary capture (Capture instance fit as a random effect). In all plots, dashed vertical lines correspond to 9.2 and 10.6 h, the times at which break point analysis detected a changepoint in mobility following tagging and secondary capture, respectively. The points in C correspond to raw IVM values used to fit the model.

**FIGURE 5:**
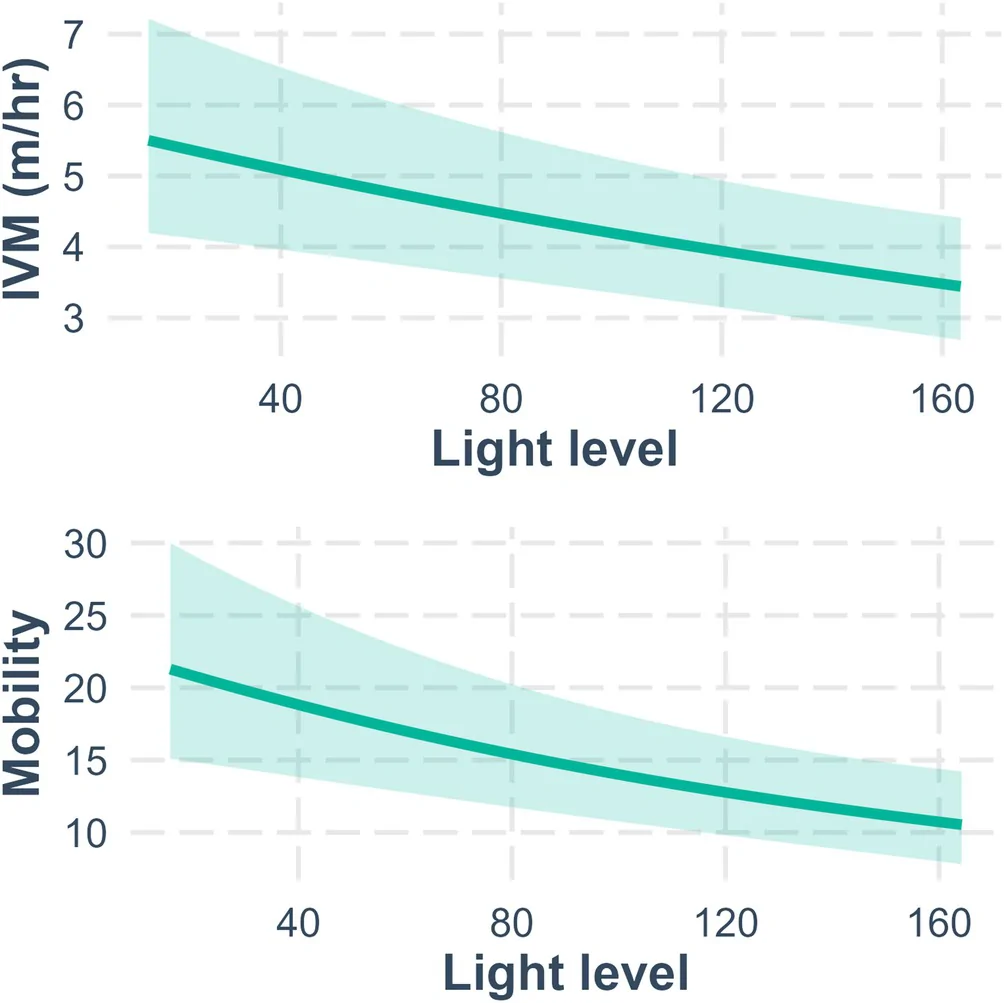
Effect of light level on the integrated vertical movement (IVM) and mobility (based on dynamic acceleration) of the tagged flapper skate, modelled using two separate generalised additive models (GAMs).

Change point analysis identified a decrease in mobility at 9.2 h and 10.6 h following the first and second captures, respectively. These timings also correspond with the skate's depth settling at close to the median depth across the full tagged duration (Figure 4a). Outputs from the GAMs corroborate that IVM was high in the periods post‐release (Figure 4b,c), as was the mobility. The model describing IVM explained 74.8% of the deviance in this time series, while the model describing mobility explained 29.5% (Table S1). While the IVM model outperformed the mobility model, this is likely due to the hourly sample rate of 1 h for IVM, as opposed to 10 min for mobility, which leaves much more fine‐scale variability evident in the data (Table S1). While tide level did not seem to impact either IVM or mobility (Table S1), light level had a negative correlation with both (Table S1, Figure 5), suggesting that this flapper skate was more active at night.

**FIGURE 6:**
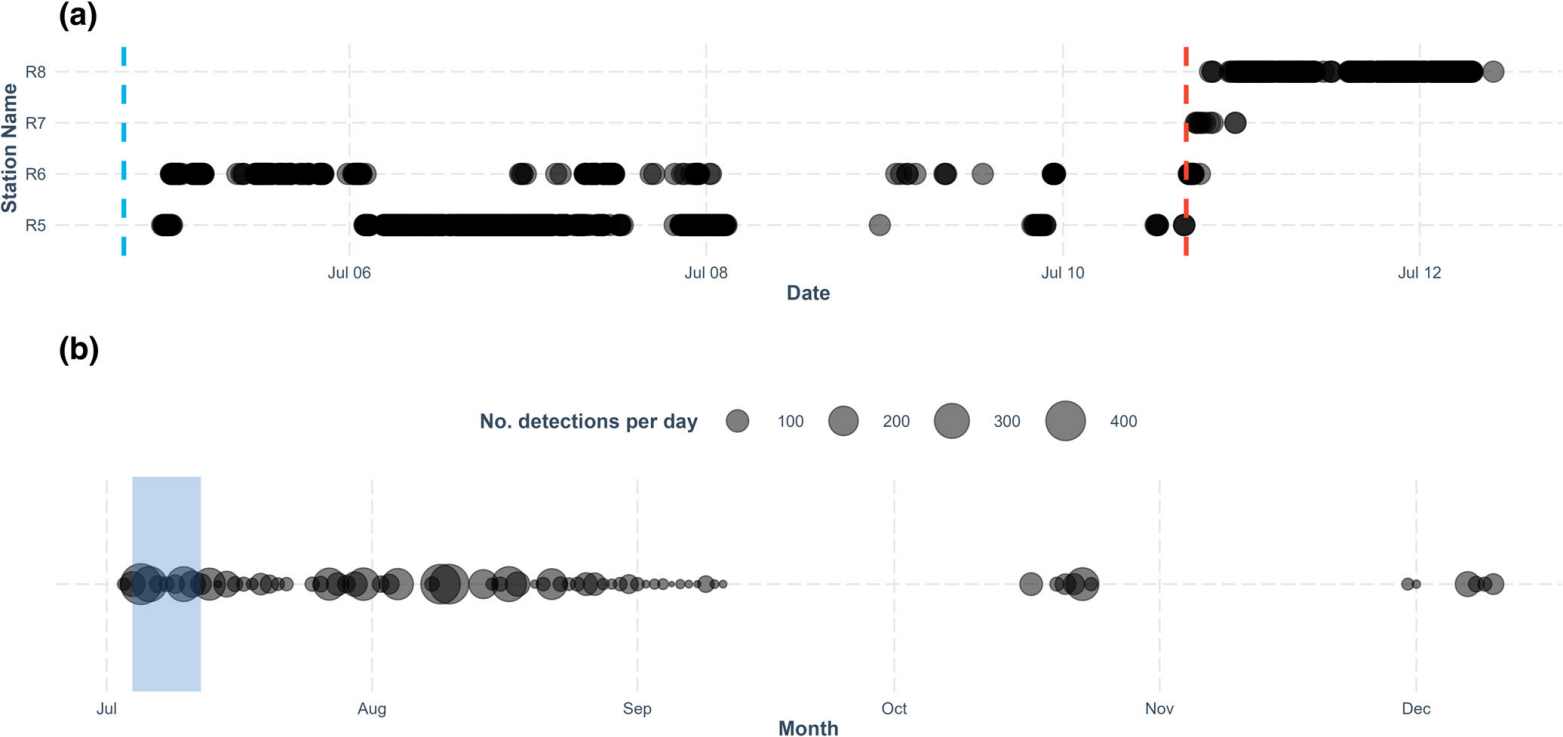
Horizontal movements of the acoustically tagged flapper skate in 2024. (A) Acoustic detections of the female flapper skate across the 7‐day satellite‐tracking period. The blue dashed line corresponds to the first capture event, and the red dashed line corresponds to the second. (B) Acoustic detections recorded across the entire acoustic tracking study period between 24th July 2024 and 8th February 2025, with the 7‐day satellite‐tracking period highlighted in light blue.

The skate was detected by the acoustic receiver array every day across the 7‐day window, with a total of 1244 unique detections recorded (daily detection counts ranged from 21 to 430) (Figure 6a). The skate was initially tagged just outside the acoustic gate (0.7 km SE of R4 and 1 km SW of R5; Figure 1) and was first detected ~6 h post‐capture on R5 (moored at 31 m). Within the 7‐day period, acoustic detections were recorded on four unique neighbouring gate receivers, covering a cumulative estimated listening area of 2 km^2^, and moored at depths of 28–31 m. During this period, the longest time at liberty (the time between successive detections) occurred almost 4 days after the initial capture event and lasted for ~21 h. For the first 6 days of the study, the skate was predominantly detected on receivers R5 and R6. After the second capture event on the 10th of July, the skate moved from the second capture site west (to R5), then east, and was sequentially detected on R5 to R8.

A further 5323 detections were recorded during the subsequent 212‐day acoustic tracking period after the satellite tag had popped off the animal, resulting in a cumulative 73 residency days across the entire 219‐day acoustic tracking study (Figure 6b). Detections declined after the summer period, with prolonged periods at liberty between mid‐September to mid‐October (~36 days), late‐October to late‐November (~37 days) and mid‐December 2024 to the end of the study period in early February 2025 (~57 days).

## 4 DISCUSSION

The short‐term response of skates and rays (Batoidea), including the critically endangered flapper skate, to catch‐and‐release (C&R) angling has received limited study to date (Cameron et al., 2023). Yet, flapper skates are routinely captured and recaptured across their remaining restricted range (Orrell et al., 2026). Using high‐resolution archival data and acoustic tracking, we present the post‐release behaviour of a single flapper skate that was captured, tagged and recaptured 6 days later. This is the first fine‐scale biologging dataset for flapper skate generated in Irish waters, and the second high‐resolution C&R biologging dataset for this species. While our study focuses on a single individual, the observed behaviours are consistent with those reported in the only other C&R flapper skate biologging dataset to date, generated by Lavender et al. (2022). Uniquely in our study, we used a high‐resolution archival tag to monitor post‐release behaviour over a short‐term study period (7 days) and acoustic telemetry to provide insights into horizontal movements during the satellite‐tracking data frame and during a longitudinal monitoring window (219 days).

### 4.1 Short‐term behavioural responses to catch‐and‐release angling

Given the nature of tracking datasets, it is typically not possible to discern the role of tagging and related procedures versus capture alone in shaping animal behaviour (Hvas et al., 2020). For example, tracking wild animals typically involves the capture, handling, tagging and often holding before release. Therefore, investigating the effects of animal capture versus tagging procedures is challenging due to the potential influence of factors such as increased time on deck, anaesthetics and procedural effects. Uniquely, the dataset presented here provides the behavioural response of a flapper skate to two separate C&R events.

The tagged flapper skate displayed a typical flight response after the first capture, leaving the proximate tagging area. However, it is unlikely to have travelled far, given it was detected 6 h later on the acoustic array. After leaving the array area, it rested on the seabed for ~1 h, indicative of recovery behaviour. Resting behaviour may relate to the physiological and biochemical effects associated with capture‐induced exhaustion (Danylchuk et al., 2014; Wells et al., 1986), or depth‐holding associated with a freeze‐and‐hide behavioural response (Shi et al., 2024). A similar 1–2 h resting recovery response was observed in rod‐and‐line‐caught flapper skate by Lavender et al. (2022) and has also been reported for other elasmobranch species. For example, a reduction in swim speeds 6 h post‐capture and handling has been reported for a range of obligate ram‐ventilating shark species (Iosilevskii et al., 2022). Using pop‐off archival transmission (PAT) tags, Campana et al. (2009) identified that blue sharks (*Prionace glauca*) caught on commercial pelagic longlines exhibited depth‐holding recovery behaviour for 2 to 7 d post‐release. After a short rest period, the tagged flapper skate exhibited high‐energy vertical movements for 8 h before a reduction in horizontal and vertical activity, remaining close to the initial capture site, enabling its subsequent recapture. After the second capture event, the skate immediately engaged in high‐energy vertical movements for ~10.6 h, before reducing its vertical and horizontal activity and returning to its ‘typical’ depth recorded before the second capture event. Grainger et al. (2022) stipulated that the rapid tailbeats and offshore movements of white sharks (*Carcharodon carcharias*) following capture on SMART drumlines are a post‐capture “flight” response. Similarly, the observed oscillatory vertical movements of the tagged flapper skate after the second capture event and transit from the study area may reflect a flight response.

While the short‐term and long‐term responses of batoids to C&R angling remain uncertain, mark–recapture data suggest high survivorship (Régnier et al., 2024). Physiological and biologging studies suggest that skates exhibit a short‐term behavioural response to capture (Cole et al., 2024; Lavender et al., 2022). Lavender et al. (2022) found that 71% of tagged flapper skate (*n* = 15) exhibited a short period (~1–2 h) of minimal vertical activity following release, and overall, the average vertical activity of tagged skates was ~38% higher in the 12 h following release compared to undisturbed activity. Similarly, the individual studied here exhibited increased mobility and vertical movements, followed by a decrease at 9.2 h and 10.6 h post‐capture. The average post‐capture IVM in our study is higher than that reported by Lavender et al. (2022) at 441%.

Outside the capture–recovery window, the tagged skate showed minimal vertical activity, with some suggestion that they are generally more active at night. This is an important insight into the animal's activity patterns, while also providing a case for including time since capture in biologging data streams to mitigate against the confounding impact of capture on movement features. Wearmouth & Sims (2009) also identified that satellite‐tagged female skate (*n* tagged individuals = 6, TL range = 1.3–2.0 m) exhibited stationary behaviour with brief periods of vertical activity, thought to be ambush feeding or a response to disturbance. This study identified that a female common skate (TL = 1.32 m) spent periods of up to 30 h (more than two tidal cycles) resting on the seabed. Similar resting behaviour has been observed in other dorsoventrally flattened species (Gibson, 2005; Humphries et al., 2017).

A range of factors have been linked to C&R behavioural responses and survival. For example, Cole et al. (2024) identified that elevated water temperatures and fish fight times increase the physiological stress experienced by rod‐and‐line‐caught flapper skate. The temperature recorded on the satellite tag during the 7‐day fine‐scale tracking window increased from ~12°C on the initial day of capture to 13–14°C on the day of the second capture event. This substantial increase in water temperature during this short‐term study highlights that animals captured only days apart may experience variable physiological stress associated with elevated temperature.

Additionally, fish fight times and time spent on deck were longer after the first capture event (8 and 18 min, respectively) than after the second (3 and <5 min, respectively). The skate did not exhibit a ‘resting’ response after the second capture, as observed after the initial capture. This lack of a static period before engaging in high‐energy vertical movements may suggest the skate was not exhausted, which is likely given the 3‐min fight time. In support of this, the skate took longer to return to lower activity levels after the second capture event (10.6 h) than after the first (9.2 h).

### 4.2 Residency

The skate was recorded on the acoustic array every day within the 7 day tracking period, confirming that it remained close to the capture site. Based on the incidental recapture of the tagged individual 6 days later, it seems unlikely the skate became “hook shy” as observed in other large fish species (Griffin et al., 2024). As also suggested by the multiple recaptures of conventionally tagged individuals recorded in flapper skate mark‐recapture datasets (Neat et al., 2015; Orrell et al., 2026). The flapper skate tracked here was detected for one‐third of the entire 219 day tracking period, with a decline in the number of acoustic detections after the summer period. Similarly, Lavender et al. (2021) found that acoustically tagged immature female flapper skate exhibited either short‐term (*n* = 4) or long‐term (*n* = 2) residency, with both immature and mature females detected more frequently after tagging in spring/summer. Given the acoustic gate was deployed exclusively in Courtmacsherry Bay, it is not possible to determine whether it truly left the proximate area or simply moved >400 m away. Detections were recorded on four receivers in proximity to slightly deeper mud channels, which are known habitats and areas for *Nephrops* (as targeted by local boats; D. Orrell *pers. comm*.). *Nephrops* are thought to be one of the predominant prey items for the flapper skate (Dulvy et al., 2006). Therefore, periodic residency may be driven by feeding, which may also explain the observed bursts in mobility. However, from tracking data alone, it is not possible to definitively determine the drivers of residency and space use.

### 4.3 Potential management implications and directions for future work

This dataset provides insight into the fine‐scale movement behaviour of an animal equipped with a high‐resolution archival tag, enabling the behavioural response to be recorded in relation to two separate capture events. Importantly, acoustic data indicate that while flapper skate exhibit a short‐term behavioural response to capture by angling in shallow water, they remain within the proximate area even after multiple capture events. This finding suggests that in the context of spatial management measures, flapper skate remain site‐attached despite multiple C&R angling interactions. However, further research is required to assess the behavioural and physiological effects of C&R angling, ideally across both males and females at varying stages of development and at different times of year, to identify the potential drivers of inter‐individual behavioural responses to C&R practices.

Despite two capture events, this research highlighted that the skate exhibited a short‐term behavioural response: It initially moved away from the bay after the first capture but returned to the area where it was captured again. After this second capture event, it remained within the acoustic array area. A caveat of this research is that the sample size is limited to a single individual. Given the conservation status of this species and the co‐occurrence of recreational C&R angling within existing protected areas elsewhere in its range, further research is key to understanding the behavioural and physiological impacts of C&R angling. In Ireland, freshwater and marine angling (of all fish species, including retained and C&R fish) generates an estimated €755 million annually for the Irish economy (Tourist Development International, 2013). The data gleaned from angler‐led mark–recapture flapper skate datasets to date have formed the foundation of our current understanding of flapper skate movements in both Scottish (Little, 1995; Neat et al., 2015) and Irish waters (Orrell et al., 2026). Given the value and interest in C&R angling for conservation‐listed species, including flapper skate, research is urgently needed to identify how to minimise harm and limit the behavioural impacts of this practice (Cameron et al., 2023). This information is crucial for designing effective species management plans (Arostegui et al., 2021). Therefore, additional study on the effects of angling practices on batoid species will support the development of robust guidelines and further promote animal welfare. In the context of flapper skate, while fish must be released alive, there are currently no implemented guidelines in the Republic of Ireland on specifics such as hook types, landing methods or duration beyond those provided for elasmobranchs in general (Inland Fisheries Ireland, 2022). In line with Scottish initiatives (NatureScot, 2026), a practical next step to minimise C&R interaction effects should include the co‐development and dissemination of species‐specific best‐practice guidelines co‐written by researchers, the state and the angling community. Given the reliance on existing community science mark–recapture programmes to generate essential monitoring data, teasing apart C&R effects and approaches to minimise harm are key to supporting conservation management efforts.

## AUTHOR CONTRIBUTIONS

DLO, DH, AC, LH and TD conducted the fieldwork. DLO and JD conceptualised the analysis. DLO undertook the horizontal movement analysis. JD undertook the vertical movement analysis. DLO led the writing of the manuscript with contributions from JD, TD, DH, AC and LH. DLO and TD secured project funding. All authors read and approved the final manuscript.

## FUNDING INFORMATION

This work was supported by a grant from the Sustainable Energy Authority of Ireland, 21/RDD/670. The Sustainable Energy Authority of Ireland has funded this work under the SEAI Research, Development & Demonstration Funding Programme 2021, The CETUS Project, Grant number 21/RDD/670. This is manuscript #6 of the CETUS Project. It also forms part of the Flapper Skate Project funded by the Department of Housing, Local Government and Heritage. DLO is funded by the CETUS project. JD is funded by a Horizon Europe MSCA Global Fellowship, Grant number 101148550.

## Supporting information


**Figure S1.** Detection probability as a function of distance. Hourly means of detection probability per distance interval (250, 400, 500, 600, 700 and 800 m) are visualised as black circles. The red line represents a cubic spline fit to the mean detection probability.
**Figure S2**. A MiniPAT PSAT tag was attached to a flapper skate *Dipturus intermedius*, to track its fine‐scale movement across the 7‐day study period. (A) Close‐up of the tether attachment. (B) A female flapper skate with the satellite tag affixed to the wing.
**Figure S3**. Residual tests for the generalised additive mixed‐effects model describing integrated vertical movement of a skate post‐capture. Plots relate to distributions of expected versus actual residuals (QQ plot, left), dispersion (centre) and outlier (right).
**Figure S4**. Plot of observed versus expected model residuals for the generalised additive mixed‐effects model describing mobility of a skate post‐capture. The red points correspond to typical residuals and the black points correspond to standardised residuals, accounting for the autocorrelation structure specified in the model. The diagonal line represents the theoretical path of exactly normal residuals.
**Table S1**. Model metrics and covariate metrics of generalised additive models describing mobility and IVM. Model metrics are sample size (*n*), deviance explained and autoregressive (order 1) coefficient of correlation, that is, a measure of serial autocorrelation and associated corrective term. Covariate metrics presented are estimated degrees of freedom (EDF), followed by *F*‐value in brackets. EDF is a measure of the complexity of a model term. *F*‐value describes the terms contribution to model fit. An asteriks (*) is appended to the effects with an EDF of 1 or greater and a *p*‐value of <0.05.

## Data Availability

Data and supporting code are available from https://doi.org/10.5281/zenodo.20592283.

## References

[jfb70542-bib-0001] Adams, K. R. , Fetterplace, L. C. , Davis, A. R. , Taylor, M. D. , & Knott, N. A. (2018). Sharks, rays and abortion: The prevalence of capture‐induced parturition in elasmobranchs. Biological Conservation, 217, 11–27. 10.1016/j.biocon.2017.10.010

[jfb70542-bib-0002] Arostegui, M. C. , Anderson, C. M. , Benedict, R. F. , Dailey, C. , Fiorenza, E. A. , & Jahn, A. R. (2021). Approaches to regulating recreational fisheries: Balancing biology with angler satisfaction. Reviews in Fish Biology and Fisheries, 31(3), 573–598. 10.1007/s11160-021-09662-y

[jfb70542-bib-0003] Bacheler, N. M. , Buckel, J. A. , Hightower, J. E. , Paramore, L. M. , & Pollock, K. H. (2009). A combined telemetry – Tag return approach to estimate fishing and natural mortality rates of an estuarine fish. Canadian Journal of Fisheries and Aquatic Sciences, 66(8), 1230–1244. 10.1139/F09-076

[jfb70542-bib-0004] Binstock, A. L. , Richards, T. M. , Wells, R. J. D. , Drymon, J. M. , Gibson‐Banks, K. , Streich, M. K. , & Mohan, J. A. (2023). Variable post‐release mortality in common shark species captured in Texas shore‐based recreational fisheries. PLoS One, 18, e0281441. 10.1371/journal.pone.0281441 PMC9925081 36780489

[jfb70542-bib-0005] Borucinska, J. , Kohler, N. , Natanson, L. , & Skomal, G. (2002). Pathology associated with retained fishing hooks in blue sharks, *Prionace glauca* (L.), with implications for their conservation. Journal of Fish Diseases, 25, 515–521. 10.1046/j.1365-2761.2002.00396.x

[jfb70542-bib-0006] Brijs, J. , Sandblom, E. , Axelsson, M. , Sundell, K. , Sundh, H. , Huyben, D. , Broström, R. , Kiessling, A. , Berg, C. , & Gräns, A. (2018). The final countdown: Continuous physiological welfare evaluation of farmed fish during common aquaculture practices before and during harvest. Aquaculture, 495, 903–911. 10.1016/j.aquaculture.2018.06.081

[jfb70542-bib-0007] Cameron, J. , Roche, W. , Beckett, W. K. , & Payne, N. L. (2023). A review of elasmobranch catch‐and‐release science: Synthesis of current knowledge, implications for best practice and future research directions. Conservation Physiology, 11(1), coad100. 10.1093/conphys/coad100 PMC10756054 38161598

[jfb70542-bib-0008] Campana, S. , Joyce, W. , & Manning, M. (2009). Bycatch and discard mortality in commercially caught blue sharks *Prionace glauca* assessed using archival satellite pop‐up tags. Marine Ecology Progress Series, 387, 241–253. 10.3354/meps08109

[jfb70542-bib-0009] Clarke, M. , Farrell, E. D. , Roche, W. , Murray, T. E. , Foster, S. , & Marnell, F. (2016). Ireland red list No. 11: Cartilaginous fish [sharks, skates, rays and chimaeras]. National Parks and Wildlife Service, Department of Arts, Heritage, Regional, Rural and Gaeltacht Affairs.

[jfb70542-bib-0010] Cole, G. , Lavender, E. , Naylor, A. , Girling, S. , Aleynik, D. , Oppel, S. , Dodd, J. , & Thorburn, J. (2024). Physiological responses to capture, handling and tagging in the critically endangered flapper skate (*Dipturus intermedius*). Conservation Physiology, 12, coae077. 10.1093/conphys/coae077 PMC11604123 39610408

[jfb70542-bib-0011] Cooke, S. J. , & Suski, C. D. (2005). Do we need species‐specific guidelines for catch‐and‐release recreational angling to effectively conserve diverse fishery resources? Biodiversity and Conservation, 14, 1195–1209. 10.1007/s10531-004-7845-0

[jfb70542-bib-0016] Council Regulation (EC) No43/2009 of 16 January 2009 fixing for 2009 the fishing opportunities and associated conditions for certain fish stocks and groups of fish stocks, applicable in Community waters and, for Community vessels, in waters where catch limitations are required. Document 32009R0043. Available from https://www.legislation.gov.uk/eur/2009/43/contents#. Accessed 19th June 2026.

[jfb70542-bib-0012] Danylchuk, A. J. , Suski, C. D. , Mandelman, J. W. , Murchie, K. J. , Haak, C. R. , Brooks, A. M. L. , & Cooke, S. J. (2014). Hooking injury, physiological status and short‐term mortality of juvenile lemon sharks (*Negaprion bevirostris*) following catch‐and‐release recreational angling. Conservation Physiology, 2, cot036. 10.1093/conphys/cot036 PMC4732486 27293620

[jfb70542-bib-0014] Dulvy, N. K. , Notarbartolo di Sciara, G. , Serena, F. , Tinti, F. , Ungaro, N. , Mancus, C. , et al. (2006). *Dipturus batis*. The IUCN Red List of Threatened Species 2006: e.T39397A10198950. Available online at: https://www.iucnredlist.org/species/39397/10198950. Accessed 9th September 2025.

[jfb70542-bib-0015] Ellis, J. R. , McCully‐Philips, S. R. , Sims, D. , Walls, R. H. L. , Choek, J. , Derrick, D. , & Dulvy, N. K. (2024). *Dipturus intermedius* (amended version of 2021 assessment). The IUCN Red List of Threatened Species 2024: e.T18903491A256581177. 10.2305/IUCN.UK.2024-1.RLTS.T18903491A256581177.en

[jfb70542-bib-0017] Freire, K. M. F. , Belhabib, D. , Espedido, J. C. , Hood, L. , Kleisner, K. M. , Lam, V. W. L. , Machado, M. L. , Mendonça, J. T. , Meeuwig, J. J. , Moro, P. S. , Motta, F. S. , Palomares, M. L. D. , Smith, N. , Teh, L. , Zeller, D. , Zylich, K. , & Pauly, D. (2020). Estimating global catches of marine recreational fisheries. Frontiers in Marine Science, 7(12). 10.3389/fmars.2020.00012

[jfb70542-bib-0018] French, R. P. , Lyle, J. , Tracey, S. , Currie, S. , & Semmens, J. M. (2015). High survivorship after catch‐and‐release fishing suggests physiological resilience in the endothermic shortfin mako shark (*Isurus oxyrinchus*). Conservation Physiology, 3, cov044. 10.1093/conphys/cov044 PMC4778490 27303650

[jfb70542-bib-0019] Gallagher, A. J. , Staaterman, E. R. , Cooke, S. J. , & Hammerschlag, N. (2017). Behavioural responses to fisheries capture among sharks caught using experimental fishery gear. Canadian Journal of Fisheries and Aquatic Sciences, 74(1), 1–7. 10.1139/cjfas-2016-0165

[jfb70542-bib-0020] Garbett, A. , Phillips, N. D. , Houghton, J. D. R. , Prodöhl, P. , Thorburn, J. , Loca, S. L. , Eagling, L. E. , Hannon, G. , Wise, D. , Pothanikat, L. , Gordon, C. , Clarke, M. , Williams, P. , Hunter, R. , McShane, R. , Brader, A. , Dodd, J. , McGonigle, C. , McIlvenny, H. , … Collins, P. C. (2021). The critically endangered flapper skate (*Dipturus intermedius*): Recommendations from the first flapper skate working group meeting. Marine Policy, 124, 104367.

[jfb70542-bib-0021] Gibson, R. N. (2005). The behaviour of flatfishes. In Flatfishes (pp. 213–239). John Wiley & Sons, Ltd. 10.1002/9780470995259.ch10

[jfb70542-bib-0022] Grainger, R. , Raubenheimer, D. , Peddemors, V. M. , Butcher, P. A. , & Machovsky‐Capuska, G. E. (2022). Integrating biologging and behavioral state modeling to identify cryptic behaviors and post‐capture recovery processes: New insights from a threatened marine apex predator. Frontiers in Marine Science, 8, 791885. 10.3389/fmars.2021.791185

[jfb70542-bib-0023] Griffin, L. P. , Fordham, G. , Curd, G. , Adam, P. A. , Narty, C. , Rose‐Innes, K. , Merwe, D. V. , Brighton, E. , & Danylchuk, A. J. (2024). Beyond the hook: Do angler‐fish interactions in a catch‐and‐release recreational fishery modify fish space use and catchability? Canadian Journal of Fisheries and Aquatic Sciences, 84, 565–571.

[jfb70542-bib-0024] Hays, G. C. , Bailey, H. , Bograd, S. J. , Bowen, W. D. , Campagna, C. , Carmichael, R. H. , Casale, P. , Chiaradia, A. , Costa, D. P. , Cuevas, E. , Nico de Bruyn, P. J. , Dias, M. P. , Duarte, C. M. , Dunn, D. C. , Dutton, P. H. , Esteban, N. , Friedlaender, A. , Goetz, K. T. , Godley, B. J. , … Sequeira, A. M. M. (2019). Translating marine animal tracking data into conservation policy and management. Trends in Ecology & Evolution, 34(5), 459–473. 10.1016/j.tree.2019.01.009 30879872

[jfb70542-bib-0025] Hays, G. C. , Bastian, T. , Doyle, T. K. , Fossette, S. , Gleiss, A. C. , Gravenor, M. B. , Hobson, V. J. , Humphries, N. E. , Lilley, M. K. , Pade, N. G. , & Sims, D. W. (2012). High activity and Lévy searches: Jellyfish can search the water column like fish. Proceedings of the Royal Society B: Biological Sciences, 279, 465–473. 10.1098/rspb.2011.0978 PMC3234559 21752825

[jfb70542-bib-0026] Hightower, J. E. , & Pollock, K. H. (2013). Tagging methods for estimating population size and mortality rates of inland striped bass populations. American Fisheries Society Symposium, 80, 249–262.

[jfb70542-bib-0027] Hoolihan, J. P. , Luo, J. , Abascal, F. J. , Campana, S. E. , De Metrio, G. , Dewar, H. , Domeier, M. L. , Howey, L. A. , Lutcavage, M. E. , Musyl, M. K. , Neilson, J. D. , Orbesen, E. S. , Prince, E. D. , & Rooker, J. R. (2011). Evaluating post‐release behaviour modification in large pelagic fish deployed with pop‐up satellite archival tags. ICES Journal of Marine Science, 68, 880–889. 10.1093/icesjms/fsr024

[jfb70542-bib-0028] Humphries, N. E. , Simpson, S. J. , & Sims, D. W. (2017). Diel vertical migration and central place foraging in benthic predators. Marine Ecology Progress Series, 582, 163–180. 10.3354/meps12324

[jfb70542-bib-0029] Hvas, M. , Folkedal, O. , & Oppedal, F. (2020). Heart rate bio‐loggers as welfare indicators in Atlantic salmon (*Salmo salar*) aquaculture. Aquaculture, 529, 735630. 10.1016/j.aquaculture.2020.735630

[jfb70542-bib-0030] Inland Fisheries Ireland . (2022). Best practice angling and handling of sharks, skates and rays. [brochure]. Inland Fisheries Ireland. https://fishinginireland.info/wp-content/uploads/2022/07/Final-Handlingx-Brochure-July-22-Web.pdf. Accessed 25th February 2026

[jfb70542-bib-0031] Iosilevskii, G. , Kong, J. D. , Meyer, C. G. , Watanabe, Y. Y. , Papastamatiou, Y. P. , Royer, M. A. , Nakamura, I. , Sato, K. , Doyle, T. K. , Harman, L. , Houghton, J. D. R. , Barnett, A. , Semmens, J. M. , Maoiléidigh, N. Ó. , Drumm, A. , O'Neill, R. , Coffey, D. M. , & Payne, N. L. (2022). A general swimming response in exhausted obligate swimming fish. Royal Society Open Science, 9, 211869. 10.1098/rsos.211869 PMC9490326 36147936

[jfb70542-bib-0032] Kane, A. , Pirotta, E. , Wischnewski, S. , Critchley, E. J. , Bennison, A. , Jessopp, M. , & Quinn, J. L. (2020). Spatio‐temporal patterns of foraging behaviour in a wide‐ranging seabird reveal the role of primary productivity in locating prey. Marine Ecology Progress Series, 646, 175–188. 10.3354/meps13386

[jfb70542-bib-0033] Lavender, E. , Aleynik, D. , Dodd, J. , Illian, J. , James, M. , Wright, P. J. , Smout, S. , & Thorburn, J. (2021). Movement patterns of a critically endangered elasmobranch (*Dipturus intermedius*) in a marine protected area. Aquatic Conservation: Marine and Freshwater Ecosystems, 32(2), 348–365. 10.1002/aqc.3753

[jfb70542-bib-0034] Lavender, E. , Aleynik, D. , Dodd, J. , Illian, J. , James, M. , Wright, P. J. , Smout, S. , & Thorburn, J. (2022). Behavioural responses of a large, benthic elasmobranch to catch‐and‐release angling. Frontiers in Marine Science, 9, 864344. 10.3389/fmars.2022.864344

[jfb70542-bib-0035] Lindeløv, J. K. (2020). Mcp: An R package for regression with multiple change points. OSF Preprints. 10.31219/osf.io/fzqxv

[jfb70542-bib-0036] Little, W. (1995). Common skate and tope: First results of Glasgow Museum's tagging study. Glasgow Naturalist, 22(5), 455–466.

[jfb70542-bib-0063] Loca, S. L. , Collins, P. C. , Garbett, A. , McGeady, R. , Thorburn, J. , & McGonigle, C. (2025). On the Brink: Mapping the Last Strongholds of the Critically Endangered Flapper Skate (*Dipturus intermedius*). Ecology and Evolution, 15(7). 10.1002/ece3.71650 PMC12209335 40599884

[jfb70542-bib-0037] Mandelman, J. W. , & Skomal, G. B. (2009). Differential sensitivity to capture stress assessed by blood acid–base status in five carcharhinid sharks. Journal of Comparative Physiology. B, 179, 267–277. 10.1007/s00360-008-0306-4 18846381

[jfb70542-bib-0038] Mawer, R. , Dodd, J. , Thorburn, J. , Burns, N. M. , & Bailey, D. M. (2025). Using citizen science photographs to identify reproductive events in an oviparous elasmobranch. Journal of Fish Biology, 107(2), 1–12. 10.1111/jfb.70044 PMC12360143 40195933

[jfb70542-bib-0039] McLoughlin, K. , & Eliason, G. (2008). Review on information on cryptic mortality and the survival of sharks and rays released by recreational fishers. Commonwealth of Australia. SEDAR21‐RD‐22. Available from. https://sedarweb.org/documents/s21rd22-review-of-information-on-cryptic-mortality-and-the-survival-of-sharks-and-rays-released-by-recreational-fishers/. Accessed January 28th 2025.

[jfb70542-bib-0040] Mohan, J. A. , Jones, E. R. , Hendon, J. M. , Falterman, B. , Boswell, K. M. , Hoffmayer, E. R. , & Wells, R. J. D. (2020). Capture stress and post‐release mortality of blacktip sharks in recreational charter fisheries of the Gulf of Mexico. Conservation Physiology, 8(1), coaa041. 10.1093/conphys/coaa041 PMC7233284 32440352

[jfb70542-bib-0041] NatureScot . (2026). NatureScot Skate Handling Guide. https://skatespotter.sams.ac.uk/guides/handling.php. Accessed 24th February 2026.

[jfb70542-bib-0042] Neat, F. , Pinto, C. , Burrett, I. , Cowie, L. , Travis, J. , Thorburn, J. , Gibb, F. , & Wright, P. J. (2015). Site fidelity, survival and conservation options for the threatened flapper skate (*Dipturus cf. intermedia*). Aquatic Conservation: Marine and Freshwater Ecosystems, 25, 6–20.

[jfb70542-bib-0043] Orrell, D. L. , Wögerbauer, C. , O'Reilly, S. , Doyle, T. K. , & Roche, W. (2026). Community science datasets identify the spatial occurrence and hotspots of flapper skate (*Dipturus intermedius*). Journal of Fish Biology, 108(2), 446–457. 10.1111/jfb.70248 PMC13052455 41065173

[jfb70542-bib-0044] R Core Team . (2024). R: A language and environment for statistical computing. R Foundation for Statistical Computing. https://www.R-project.org/

[jfb70542-bib-0045] Régnier, T. , Dodd, J. , Benjamins, S. , Gibb, F. M. , & Wright, P. J. (2021). Age and growth of the critically endangered flapper skate, *Dipturus intermedius* . Aquatic Conservation: Marine and Freshwater Ecosystems, 31(9), 2381–2388. 10.1002/aqc.3654

[jfb70542-bib-0046] Régnier, T. , Dodd, J. , Benjamins, S. , Gibb, F. M. , & Wright, P. J. (2024). Spatial management measures benefit the critically endangered flapper skate, *Dipturus intermedius* . Aquatic Conservation: Marine and Freshwater Ecosystems, 34(4), e4150. 10.1002/aqc.4150

[jfb70542-bib-0047] Salinas Ruíz, J. , Montesinos López, O. A. , Hernández Ramírez, G. , & Crossa Hiriart, J. (2023). Generalized linear models. In Generalized linear mixed models with applications in agriculture and biology. Springer. 10.1007/978-3-031-32800-8_2

[jfb70542-bib-0048] Shi, M. , Rupia, E. J. , Jiang, P. , & Lu, W. (2024). Switch from fight‐flight to freeze‐hide: The impacts of severe stress and brain serotonin on behavioral adaptations in flatfish. Fish Physiology and Biochemistry, 50, 891–909. 10.1007/s10695-024-01298-6 38308734

[jfb70542-bib-0049] Skomal, G. B. (2007). Evaluating the physiological and physical consequences of capture on post‐release survivorship in large pelagic fishes. Fisheries Management and Ecology, 14(2), 81–89. 10.1111/j.1365-2400.2007.00528.x

[jfb70542-bib-0050] Skomal, G. B. , & Mandelman, J. W. (2012). The physiological response to anthropogenic stressors in marine elasmobranch fishes: A review with a focus on the secondary response. Comparative Biochemistry and Physiology Part A: Molecular & Integrative Physiology, 162(2), 146–155. 10.1016/j.cbpa.2011.10.002 22008842

[jfb70542-bib-0061] Skubel, R. A. , Wilson, K. F. , Papastamatiou, Y. P. , Verkamp, H. J. , Sulikowski, J. A. , Benetti, D. , & Hammerschlag, N. (2020). A scalable, satellite‐transmitted data product for monitoring high‐activity events in mobile aquatic animals. Animal Biotelemetry, 8(1). 10.1186/s40317-020-00220-0

[jfb70542-bib-0052] Thorburn, J. , Cole, G. , Naylor, A. , Garbett, A. , Wilson, K. , James, M. , Dodd, J. , Houghton, J. D. R. , & Collins, P. C. (2023). Preliminary insight into the reproductive traits of the flapper skate *Dipturus intermedius* using in‐field ultrasonography and circulating hormone concentrations. Endangered Species Research, 52, 97–111. 10.3354/esr01264

[jfb70542-bib-0053] Tourist Development International . (2013). Socio‐economic study of recreational angling in Ireland. Prepared on behalf of Inland Fisheries Ireland. Available from https://www.fisheriesireland.ie/sites/default/files/2021-07/tdistudyonrecreationalangling.pdf. Accessed 28th January 2025

[jfb70542-bib-0054] Watanabe, Y. Y. , & Papastamatiou, Y. P. (2023). Biologging and biotelemetry: Tools for understanding the lives and environments of marine animals. Annual Review of Animal Biosciences, 11, 247–267. 10.1146/annurev-animal-050322-073657 36790885

[jfb70542-bib-0055] Wearmouth, V. J. , & Sims, D. W. (2009). Movement and behaviour patterns of the critically endangered common skate *Dipturus batis* revealed by electronic tagging. Journal of Experimental Marine Biology, 380, 77–87.

[jfb70542-bib-0056] Wells, R. M. G. , McIntyre, R. H. , Morgan, A. K. , & Davie, P. S. (1986). Physiological stress responses in big gamefish after capture: Observations on plasma chemistry and blood factors. Comparative Biochemistry and Physiology Part A: Physiology, 84(3), 565–571. 10.1016/0300-9629(86)90366-X 2874936

[jfb70542-bib-0057] Whitney, N. M. , Lear, K. O. , Morris, J. J. , Hueter, R. E. , Carlson, J. K. , & Marshall, H. M. (2021). Connecting post‐release mortality to the physiological stress response of large coastal sharks in a commercial longline fishery. PLoS One, 16, e0255673. 10.1371/journal.pone.0255673 PMC8443047 34525094

[jfb70542-bib-0058] Whitney, N. M. , White, C. F. , Gleiss, A. C. , Schwieterman, G. D. , Anderson, P. , Hueter, R. E. , & Skomal, G. B. (2016). A novel method for determining post‐release mortality, behavior, and recovery using acceleration data loggers. Fisheries Research, 183, 210–221. 10.1016/j.fishres.2016.06.003

[jfb70542-bib-0059] Wilmers, C. C. , Nickel, B. , Bryce, C. M. , Smith, J. A. , Wheat, R. E. , & Yovovich, V. (2015). The golden age of bio‐logging: How animal‐borne sensors are advancing the frontiers of ecology. Ecology, 96(7), 1741–1753. 10.1890/14-1401.1 26378296

[jfb70542-bib-0060] Wood, S. N. , Pya, N. , & Saefken, B. (2016). Smoothing parameter and model selection for general smooth models (with discussion). Journal of the American Statistical Association, 111, 1548–1575.

