# Supplementary material for: Quantifying post‐release behaviour of a critically endangered elasmobranch over two capture events using high‐resolution archival tag data
Source: J Fish Biol. 2026 Jun 22;109(3):1616–27. doi: 10.1111/jfb.70542 (PMC13602082; doi:10.1111/jfb.70542)
Supplement: Supplementary file 1 [file JFB-109-1616-s001.docx]

**Quantifying post-release behaviour of a Critically Endangered elasmobranch over two capture events using high-resolution archival tag data**

**Supplementary material**

Danielle L. Orrell^*^, Jamie Darby, Damien Haberlin, Alfonso Cohuo, Luke Harman, Thomas K. Doyle

***Author for correspondence**
Danielle L. Orrell - [dorrell@ucc.ie](mailto:dorrell@ucc.ie) School of Biological, Earth and Environmental Sciences, University College Cork, Distillery Fields, North Mall, Cork, Ireland. MaREI, The SFI Research Centre for Energy, Climate and Marine, Beaufort Building, Environmental Research Centre, University College Cork, Ringaskiddy, Cork, Ireland. ORCID ID: 0000-0002-9449-3920.

**S1. Range testing**

A 24-hour range test was conducted between 1^st^ and 2^nd^ June 2023 off Garretstown, County Cork, Ireland (51.642°N, -8.582°W) to assess detection range and determine receiver spacing. A V16 range test tag (10 s fixed transmission rate at 152 dB re 1µPa @ 1m, Innovasea Inc., NS, Canada) was attached to an 8 mm polypropylene line anchored with a 40 kg chain anchor and terminating in an 11” trawl float with a 6” trailing buoy (to aid with recovery). The tag sat 8 m above the substrate. Six acoustic receivers (VR2W) were deployed at fixed distances of 250 m, 400 m, 500 m, 600 m, 700 m and 800 m from the tag line. A receiver line comprised of an 11” trawl float and a 6” trailing buoy. The trawl float was attached to a 14 mm polypropylene line, with the receiver attached 8 m above the substrate and the line terminating in a 50 kg chain anchor. Receiver mooring line anchor depths ranged from 20 to 26m.

The equipment was deployed between 12:20 on 1^st^ June 2023 and retrieved at 12:53 on 2^nd^ June 2023. Data was subsequently trimmed from 12:45 on 1^st^ June 2023 to 12:45 on 2^nd^ June 2023. Data manipulation and analysis were conducted in R (v. 4.4.2). Detection probability (%) was calculated per hour, and as a mean value for each fixed distance based on the expected versus the observed number of detections (Fig. S1). Based on these calculations, a conservative 400 m detection range was estimated. Thereafter, receivers were placed 800 m apart to form a gate across Courtmacsherry Bay, Co. Cork.

Figure S1. Detection probability as a function of distance. Hourly means of detection probability per distance interval (250 m, 400 m, 500 m, 600 m, 700 m and 800 m) are visualised as black circles. The red line represents a cubic spline fit to the mean detection probability.

**S2. Satellite tag attachment**

The satellite tag tether comprised a small titanium dart (45 mm length, 14 mm diameter, 3g in air; Wildlife Computers, Redmond, WA) crimped onto a 200 lb monofilament tether, which was then threaded through the base of the satellite tag and crimped (Figure S2). The tether length was kept short (approx. 1.5 cm) to minimise tag movement, reduce drag, and promote tag retention. As per recommendations from Wildlife Computers, the dart and first crimp were inserted into the dorsal musculature. Therefore, stainless steel crimps were selected owing to the biocompatible nature of stainless steel implants compared with other crimp metals. A 1 cm incision was made with a scalpel (size 22 blade) to reduce friction as the dart was applied using a handheld tag pole with an attached small titanium applicator pin with a slotted tip (which couples with the dart to maintain its position during insertion). The tagging site was selected because it was located away from the fringe of the skate wing and also away from the animal’s internal organs. While we were encouraged to apply lidocaine prior to the incision and tag insertion, it is unlikely that this provided preoperative relief; however, it may provide relief post-recovery. The dart was inserted at a depth of less than 1 cm to ensure it did not break the anterior surface of the wing while positioned below the skin to promote tag retention. The incision was closed with a single, independent, sterile suture (3-0 monofilament absorbable, Ethicon Ltd., Livingston, UK) to minimise movement of the tether and increase tag retention.

Figure S2. A MiniPAT PSAT tag was attached to a flapper skate *Dipturus intermedius,* to track its fine-scale movement across the 7-day study period. A. Close-up of the tether attachment. B. A female flapper skate with the satellite tag affixed to the wing.

**S3.	Generalised additive models**

Generalised additive models were primarily used to investigate the effect of capture on integrated vertical movement (IVM) and mobility metrics. Models were fit with a Gamma distribution with a log link, which approximates an exponential error distribution, as no native exponential family can be specified in the “mgcv” package (Wood, 2016). This error structure was subsequently found to suitably describe the errors of residuals for the IVM model without evidence of residual dispersion or violation of model assumptions (Figure S3).

Figure S3. Residual tests for the generalised additive mixed-effects model describing integrated vertical movement of a skate post capture. Plots relate to distributions of expected versus actual residuals (QQ plot, left), dispersion (center), and outlier (right).

Mobility was a response variable with a high near-zero-mass and highly autocorrelated response variable and larger sample size (Table S1). Because of this, variance of the response outweighed the variance explained by the relevant fitted model and issues of non-normality are seen in the QQ-plot of observed versus expected residuals (Figure S4). This is corrected somewhat when residuals are standardised according to the autocorrelation structure present, and the distribution of residuals approximates normal when that correction is applied.

Figure S4. Plot of observed versus expected model residuals for the generalised additive mixed-effects model describing mobility of a skate post-capture. The red points correspond to typical residuals and the black points correspond to standardised residuals, accounting for the autocorrelation structure specified in the model. The diagonal line represents the theoretical path of exactly normal residuals.

Multicollinearity between fitted covariates was tested using the *concurvity* function in the *mgcv* package, and no issues of high multicollinearity were detected (values < 0.7).

Table S1. Model metrics and covariate metrics of generalised additive models describing mobility and IVM. Model metrics are sample size (n), deviance explained, and autoregressive (order 1) coefficient of correlation, i.e., a measure of serial autocorrelation and associated corrective term. Covariate metrics presented are estimated degrees of freedom (EDF), followed by F-value in brackets. EDF is a measure of the complexity of a model term. F-value describes the term’s contribution to model fit. An asteriks (*) is appended to the effects with an EDF of 1 or greater and a p-value of < 0.05.

| *Model* | n | Deviance explained | AR(1) coefficient of correlation | Explanatory Covariate | | |
| --- | --- | --- | --- | --- | --- | --- |
|  |  |  |  | Time since capture | Light level | Tide |
| *Mobility* | 1021 | 29.5% | 0.61 | 7.6 (2.0)* | 1.0 (1.7)* | 0.2 (0.2) |
| *IVM* | 174 | 74.8% | 0.32 | 10.2 (5.5)* | 1.0 (1.4)* | 0.0 (0.0) |

**References**

Wood S.N., N. Pya and B. Saefken (2016) Smoothing parameter and model selection for general smooth models (with discussion). Journal of the American Statistical Association 111:1548-1575
